# Non-volatile photonic-electronic memory via 3D monolithic ferroelectric-silicon ring resonator

**DOI:** 10.1038/s41377-024-01625-9

**Published:** 2024-09-26

**Authors:** Hang Chen

**Affiliations:** Department of Electronic Engineering, Tsinghua University, Beijing, 100084 China

**Keywords:** Nanophotonics and plasmonics, Nanophotonics and plasmonics

## Abstract

A novel non-volatile photonic-electronic memory, 3D integrating an Al-doped HfO_2_ ferroelectric thin film onto a silicon photonic platform using fully compatible electronic and photonic fabrication processes, enables electrically/optically programmable, non-destructively readable, and multi-level storage functions.

Photonic computing represents one of the most promising solutions to address the exponential growth in data scale and computational demands driven by artificial intelligence in the post-Moore era^1–3^. Photonic Integrated Circuits (PICs) offer intrinsic advantages, including low latency, high parallelism, and strong immunity to electromagnetic interference^4^. By utilizing complementary metal-oxide semiconductor (CMOS) manufacturing techniques, PICs are expected to facilitate low-cost, high-volume production, positioning them as a significant breakthrough direction in the field of photonic computing^5^.

Non-volatile photonic-electronic memory stands as a critical yet challenging fundamental device for achieving PIC compatibility in photonic computing. Conventional actively tuned electro-optic modulators, including Mach-Zehnder^6^ and micro-ring resonator modulators^7^, lack non-volatility. Chalcogenide phase change materials (PCM), such as Ge_2_Sb_2_Te_5_ (GST) have garnered significant interest recently for non-volatile reversible storage^8,9^. However, the stochastic nature of crystal nucleation limits GST’s crystallization speed. Additionally, the low extinction ratio presents another challenge to its broader use.

In a recently publication in *Light: Science & Applications*, Xiao Gong’s team from National University of Singapore introduced a novel approach for non-volatile photonic-electronic memory, integrating an Al-doped HfO_2_ (HAO) ferroelectric (FE) thin film onto a silicon photonic platform as shown in Fig. 1 (ref. ^10^). The 3D monolithic integration on the silicon waveguide enables zero-energy retention of optical information. Electrical programming/erasing are controlled via E_program/erase_ port applied to the FE, while optical programming/erasing is facilitated by two photodiodes make this dual-mode operation allows simultaneous, non-destructive readout. The design places the FE thin film atop the waveguide, safeguarding other photonic components during fabrication. This approach ensures simplicity, scalability, and full compatibility with both electronic and photonic manufacturing processes.

**Fig. 1:**
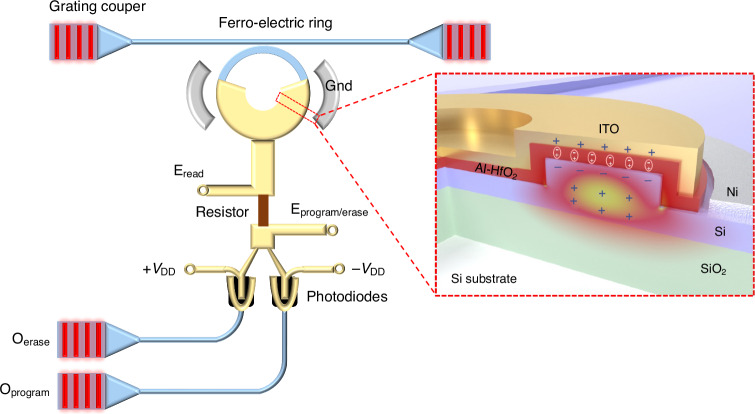
The 3D schematic of the proposed non-volatile photonic-electronic memory and its enlarged structural view

The non-volatile photonic-electronic memory exhibited strong performance in both simulations and experimental verification. Employing a ring resonator structure, the memory cell achieved an optical extinction ratio of 6.6 dB at a low operating voltage of 5 V. Retention and endurance tests confirmed its non-volatile characteristics and reliability. The device was programmed and erased using ±5 V pulses, with read operations conducted at 1 V across intervals ranging from 1 s to 1000 s at room temperature, demonstrating no significant degradation. Fitting analysis indicates an estimated retention exceeding 10 years. The memory cell demonstrated a minimum endurance of 4 × 10^4^ cycles at 5 V and 1 × 10^6^ cycles at 4 V. By leveraging the ferroelectric material’s capacity to control induced polarization via external voltage bias, the memory cell efficiently supports multi-level storage.

While advancements in PICs, particularly in non-volatile photonic-electronic memories, are driving progress in photonic computing, the realization of artificial general intelligence (AGI) demands not only device innovation but also coordinated advancements across multiple domains, including algorithms, systems, and applications. Cutting-edge algorithms, such as in-situ training^11^ and dual adaptive training^12^ methods, facilitate real-time correction of time-varying system errors through online learning, contributing to the development of efficient and energy-saving PICs. The integration of on-chip diffraction and interference opens avenues for the creation of large-scale diffractive-interference hybrid photonics chiplets^13^, fostering novel PIC systems with enhanced scalability and computational power. The scope of photonic computing is expanding beyond initial applications in simple handwritten digit classification^14^ to encompass more complex tasks, such as visual information processing^15^ and content generation^16^. These developments will also provide crucial insights for the future evolution of PICs.

## References

[1] Lin, X. et al. All-optical machine learning using diffractive deep neural networks. *Science* **361**, 1004–1008 (2018). 30049787 10.1126/science.aat8084

[2] Chen, H. et al. Diffractive deep neural networks at visible wavelengths. *Engineering* **7**, 1483–1491 (2021).

[3] Shen, Y. C. et al. Deep learning with coherent nanophotonic circuits. *Nat. Photonics* **11**, 441–446 (2017).

[4] Zhou, W. et al. Fabrication and integration of photonic devices for phase-change memory and neuromorphic computing. *Int. J. Extrem. Manuf.* **6**, 022001 (2024).

[5] Luo, W. et al. Recent progress in quantum photonic chips for quantum communication and internet. *Light Sci. Appl.* **12**, 175 (2023). 37443095 10.1038/s41377-023-01173-8 PMC10345093

[6] Zhao, H. Q. et al. Integrated preparation and manipulation of high-dimensional flying structured photons. *eLight* **4**, 10 (2024).

[7] Xu, S. F. et al. Analog spatiotemporal feature extraction for cognitive radio-frequency sensing with integrated photonics. *Light Sci. Appl.* **13**, 50 (2024). 38355673 10.1038/s41377-024-01390-9 PMC10866915

[8] Wei, M. L. et al. Electrically programmable phase-change photonic memory for optical neural networks with nanoseconds in situ training capability. *Adv. Photonics* **5**, 046004 (2023).

[9] Meng, J. W. et al. Electrical programmable multilevel nonvolatile photonic random-access memory. *Light Sci. Appl.* **12**, 189 (2023). 37528100 10.1038/s41377-023-01213-3 PMC10393989

[10] Zhang, G. et al. Thin film ferroelectric photonic-electronic memory. *Light Sci. Appl.* **13**, 206 (2024). 39179550 10.1038/s41377-024-01555-6 PMC11344043

[11] Zhou, T. K. et al. Large-scale neuromorphic optoelectronic computing with a reconfigurable diffractive processing unit. *Nat. Photonics* **15**, 367–373 (2021).

[12] Zheng, Z. Y. et al. Dual adaptive training of photonic neural networks. *Nat. Mach. Intell.* **5**, 1119–1129 (2023).

[13] Chen, H. & Shen, Y. C. Large-scale distributed diffractive-interference hybrid photonic chiplets. *Adv. Photonics* **6**, 040502 (2024).

[14] Zheng, M. J. et al. Optimize performance of a diffractive neural network by controlling the Fresnel number. *Photonics Res.* **10**, 2667–2676 (2022).

[15] Yan, T. et al. Fourier-space diffractive deep neural network. *Phys. Rev. Lett.* **123**, 023901 (2019). 31386516 10.1103/PhysRevLett.123.023901

[16] Xu, Z. H. et al. Large-scale photonic chiplet Taichi empowers 160-TOPS/W artificial general intelligence. *Science* **384**, 202–209 (2024). 38603505 10.1126/science.adl1203

